# Performance Evaluation of Dental Flosses Pre- and Post-Utilization

**DOI:** 10.3390/ma15041522

**Published:** 2022-02-18

**Authors:** Adrian K. Stavrakis, Sanja Kojić, Bojan Petrović, Isidora Nešković, Goran M. Stojanović

**Affiliations:** 1 Faculty of Technical Sciences, University of Novi Sad, 21000 Novi Sad, Serbia; sanjakojic@uns.ac.rs (S.K.); sgoran@uns.ac.rs (G.M.S.); 2 Department of Pediatric and Preventive Dentistry, Faculty of Medicine, Dentistry Clinic of Vojvodina, University of Novi Sad, 21000 Novi Sad, Serbia; bojan.petrovic@mf.uns.ac.rs (B.P.); isidora.neskovic@mf.uns.ac.rs (I.N.)

**Keywords:** dental floss testing, plaque management, in vitro characterization, scanning electron microscopy

## Abstract

Dental floss is an oral hygiene product used to remove food and plaque in places where toothbrushes cannot reach. Even though over the years since its introduction some research in suitable materials has been performed, thread cracking and wear can still compromise efficiency. The aim of this study was to examine the morphological properties of four different commercially available dental floss types before and after use. For that purpose, scanning electron microscopy and optical microscopy were used to assess the flosses before and after use, and tension testing was performed to determine any degradation in the floss performance after utilization. The analyzed floss samples verify the hypothesis that the properties of the floss need to be known in depth, before recommending a specific type to patients for daily use in all clinical indications.

## 1. Introduction

Dental floss has long been used to clean the spaces between teeth and as a dental cleaner under fixed bridges. The inventor of dental floss, Levi Parmly (1790–1859), was the first to express concern about the cleaning of approximal surfaces [1]. He explained that the cleaning device should be “passed through the interstices of the teeth, between their necks and the arches of the gums, to dislodge that irritating matter which no brush can remove, and which is the true source of distress”.

Even though silk floss has been used in dentistry since the 19th century, and nylon floss was introduced to the market shortly after World War II, the difficulty of flossing is one of the reasons why it still is not widely used or universal, with a routine use ranging between 10–30% of adults [2,3]. Nylon, also known as polyamide (PA), is the second most common synthetic polymer used in textiles. It is one of the most versatile thermoplastics used today due to properties such as excellent strength, high Young’s modulus, stiffness, toughness, lubricity, temperature, fatigue, and abrasion resistance [4,5].

Today, a variety of flosses is commonly available. Generally, they can be categorized in monofilaments and multifilaments, waxed and unwaxed, synthetic and natural. Monofilament flosses contain only one strand of the material, while their multifilament counterparts contain several strands twisted together. Natural floss is made of silk, whereas synthetic floss is made of nylon or polytetrafluoroethylene (PTFE). The advantage of nylon is that it is incredibly low friction while relatively durable. PTFE, on the other hand, is slightly more tolerant to elongation. Wax coating is usually synthetic and petroleum-based, while some natural options with candelilla wax coating exist. Lastly, floss tapes also exist, which are generally perceived as more convenient by people with large teeth surface areas. Instead of the standard, tubular shape of the flosses, dental tapes are flattened and rectangular in cross-section.

However, the difficulty of flossing does not pertain to the material choice, even though a correct floss can potentially ease it. There are a few parameters that impact the process, although the most notable ones are the flossing technique and the existence of tight contact points in the oral cavity, which combined may lead to floss breakage, fraying, or shredding. Moreover, the subject needs to effectively reach all sextants in order for flossing to be of help towards plaque management, which is rarely the case [6]. The manual abilities of users significantly affect the quality of flossing and cleaning. It is very important that the user is well educated by the dental professional on how to properly use dental floss.

By modern standards, dental floss is typically made up of a yarn of numerous fine filaments (individual fiber diameter is around 20–30 µm) made of nylon or polyester, held together in the yarn by a binder [7]. It is further defined by ISO 28158:2010 and the revised ISO 28158:2018 as: multiple filaments gathered into thread, spun yarn, single filament, or tape, commonly synthetic fiber, with or without coating material(s), designed for the removal of plaque, or debris, or both, from the proximal surfaces of natural or artificial teeth and the gingival surfaces of bridges or fixed prostheses [8,9]. According to this standard, when a visual inspection is required, a floss sample with a holder should be examined under x10 magnification, and the integrated dental floss and handle must not have any sharp surfaces, burr, or parts except if a part is included and designed to be used as a toothpick. When used in conjunction with tooth brushing, dental floss is proven to remove plaque more effectively than toothbrushing alone [10,11].

In spite of the fact that dental floss nowadays comes in a variety of materials, including silk, nylon, and PTFE with or without wax, little is known about its physical properties, tensile strength, and structural and morphological characteristics. The majority of the information is provided by the manufacturer and is typically used for commercial claims. Combining data on structural properties, the morphology of breakdown and wear of various types of dental floss can be used to create a database for future development of dental floss products with desirable properties.

This claim can be further backed by the current market trends in the area of oral health care products. From 1992 to 2002, there was a 38.3 percent increase in the consumption of toothpaste, 138.3 percent increase in that of toothbrushes, and 177.2 percent increase in that of dental floss [12]. According to Euromonitor International, during the last 15 years (2006–2020), the expenditures for oral health care products (excluding electric toothbrushes) increased from 1180.06 to 2274.4 RSD million in Serbia, while globally, the same source reports the increase from 28,713.8 to 42,553.1 USD million [13,14].

This increase by more than 90% in Serbia and more than 40% globally, is mainly driven by awareness towards dental hygiene and primarily plaque management, as brushing alone can remove about 60% per session. Thus, a multitude of tools such as flosses, interdental brushes, and oral irrigators have been developed. Studies have shown the potential of flossing in plaque removal, but also the high likelihood that it is not used properly [6].

Dental floss did not outperform other interdental cleaning devices and was found to be less effective than interproximal brushes when both were used in conjunction with manual toothbrushing with toothpaste [15]. The latter had better bleeding and brush scores, and are associated with more plaque removal [16,17] as well as better patient acceptance.

Although interdental brushes can obviously only be used in larger interproximal spaces, studies have found no significant difference between interproximal toothbrushes and dental flosses in such spaces. A Cochrane review published in 2011 found “weak and very unreliable evidence” that flossing as a supplement to brushing may be linked to a small reduction in plaque, though they did find a significant benefit in reducing gingivitis [18]. These findings are in line with a 2015 meta-review, which found that the majority of available studies fail to demonstrate the effectiveness of flossing in plaque removal, possibly due to technical difficulties or a lack of patient compliance [19].

According to some national population surveys, one-third of the general population (34%) uses dental floss, while only 18% of the population uses interdental brushes, down from 42 percent in 2010. Given the popularity of dental floss but its low efficacy in these spaces, the development of a dental floss with ellipsoidal floss knots at regular distances has been proposed to facilitate plaque removal in wider interproximal spaces [20,21,22]. Traditional dental floss, on the other hand, has been linked to infections such as peri-implantitis, which can lead to dental implant failure [23]. This is because dental floss, especially when used around a dental implant, is prone to shredding. When dental floss shreds, it leaves particles or pieces behind that can become trapped between the implants and gum tissue, resulting in an infection.

It is still unclear what components or characteristics of dental flosses or the technique of flossing are responsible for plaque removal efficacy and undesirable effects. Generally, a subject should floss by wrapping an adequate quantity of floss around their index fingers and by using their thumb they should guide it below the contact point of their teeth, into the gums. Then, by forming it in a “C” shape around the tooth they should lift up a few times, in order to drive plaque up and out of the gingival sulcus. They should proceed regularly to a new section of floss as it traps particulates constantly. However, the subject’s familiarity, ease, and skills with flossing, as well as their oral cavity peculiarities, might cause them to deviate from the proper method and adopt their own.

There are speculations whether the waxed or coated dental floss was the cause of the host’s inflammatory response, which led to alveolar bone loss over time, or whether it was a contributing factor to an inflammatory condition that already existed. A hypersensitivity immune response in the host to one or more of the waxed or coated floss ingredients is thought to be the cause of both hypotheses. More research is needed to determine which components of waxed or coated dental floss can cause generalized refractory chronic periodontitis. Oral hygiene products, particularly waxed or coated dental floss, can cause hypersensitivity reactions, which clinicians should be aware of. A list of ingredients in commercially available dental floss would help identify potentially harmful ingredients.

Usually, the mechanical properties of dental floss are evaluated in relation to the ultimate tensile strength and elongation [24]. The ultimate tensile strength of a material determines the maximum force it can withstand before failing or breaking. The maximum elongation of the length divided by the original length is determined by the percentage of elongation. The percentage of elongation and tensile strength are important clinically in determining the mechanical properties of dental floss. The tensile strength of a material determines its durability, and the maximum load that dental floss can withstand when passing between teeth percentage of the total length to which dental floss can be stretched before it tears is determined by its elongation.

There are no data in the literature about the pattern of dental floss cracking and breakdown, so the morphology of cracking is still unknown. In relation to knowledge of the morphological characteristics during breakdown, the answer to the question of whether it breaks on sharp edges, serrated or in fragments, can provide answers about potential damage to oral structures and dental restorations when flossing.

The aim of this study was to evaluate the maximum tensile force, the percentage of elongation, and the morphology of cracking of four commercially available dental flosses before and after use.

## 2. Materials and Methods

The aim of this work is to evaluate the performance of commercially available dental flosses, and to potentially detect any major degradation in their properties after use.

In order to keep our study as close to the real conditions as possible, we chose to not force a standardized floss length on the study participants, so that they would be able to floss using their preferred method, and thus replicate as accurately as possible their everyday conditions, regardless of the fact that the output was intended for a study.

**Figure 1:**
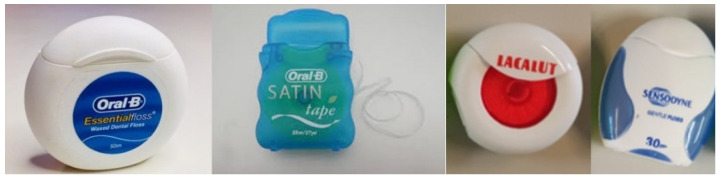
The four utilized flosses of the study in their original containers.

For the needs of this experiment, four dental flosses were utilized. These flosses are commercially available, and among the most widely used in Serbia. These flosses are Oral B Essential, Oral B Satin, Lacalut, and Sensodyne (Figure 1).

Ten participants were selected for this study, each of whom was tasked to create ten samples of each of the dental flosses by using them on their teeth at their convenience. All candidates for the study were informed before a task was assigned to them on the nature of the task, the requested mode of work, and the way that their samples would be further processed in the scope of this study, in line with the ethical approval by the Ethical Committee of Dental Clinic, Faculty of Medicine, University of Novi Sad (N° 01-21/5-21) and consented.

All collected samples were sterilized using exposure to germicidal UV-C radiation (Medivent, Foshan, China) for 60 min and subsequently refrigerated. Even though UV-C can cause alterations to polymeric surfaces in the long run [25,26], in this case, a relatively short exposure time was used, and no visual changes in color were observed, indicative that this process does not impact the integrity of the flosses. This step was carried out as normally used flosses are disposed of, but in this case, they were to be examined further. To study the performance degradation of the flosses, three methods were deployed. Firstly, optical microscopy and scanning electron microscopy (SEM) were utilized to compare a set of unused flosses to the used specimens. Such an approach has been used previously by the authors with favorable results [27]. Optical microscopy was performed using an HRM-300 upright optical microscope (Huvitz, Anyang, Korea), supported with an automated z-axis focus-module and Panasis software for making visual 3D models. To obtain focused images of the dental flosses, a 2D view was extracted from the 3D acquired optical profile. Scanning electron microscopy was performed using a TM3030 benchtop microscope (Hitachi, Tokyo, Japan). This technique has been used to depict, in more detail, the flosses and their defects.

**Figure 2:**
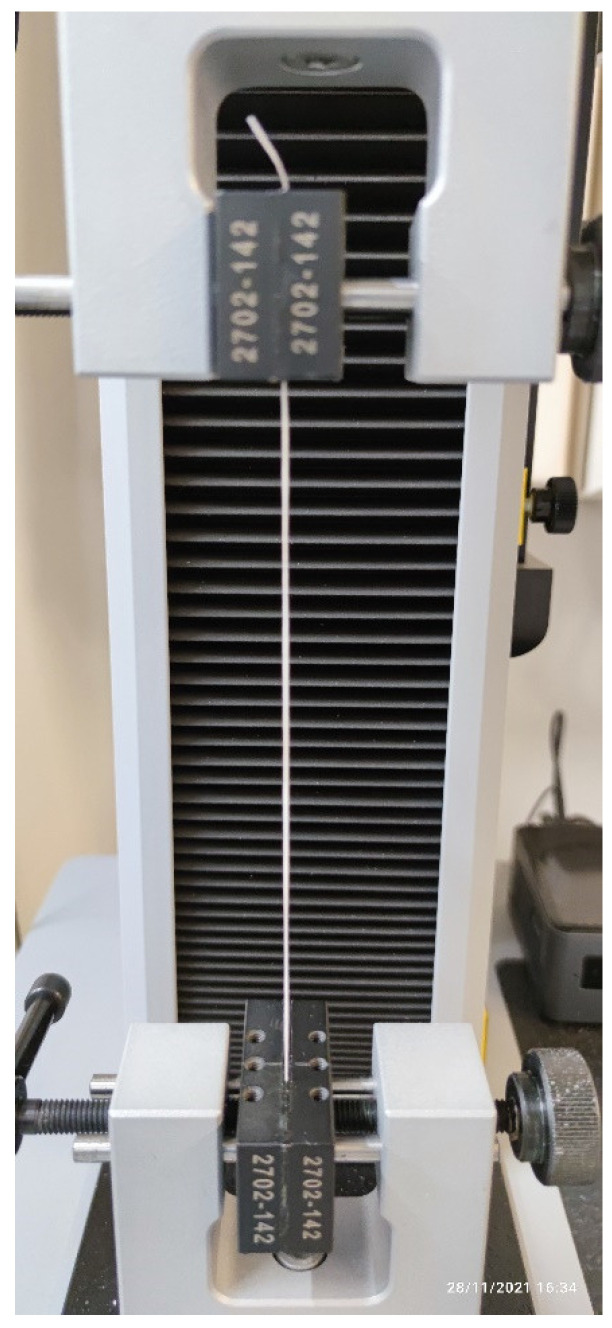
A dental floss sample loaded onto the tension testing system. The bottom part is fixed and the top part connected to a force transducer, and movable. In this case, straight, rubberized fixtures were used to avoid floss slippage.

Secondly, a tension testing machine was utilized to study the force at break and elongation both the unutilized and the utilized samples could sustain. In this study, a single-column model 34SC-2 tension testing system was used (Instron, Norwood, MA USA), with a maximum force transducer capacity of 2 kN. To attach the samples, manual rectangular grips were selected, and to avoid both floss slippage, and to damage the flosses at the grip edge, non-serrated, rubber-coated grip plates were attached. Figure 2 showcases a mounted specimen on the instrument.

To test the flosses, a custom testing method was created in the tension testing machine software. A set of 10 specimens were programmed to form a sample, as that was the number of specimens acquired per volunteer and per floss. The method instructed the instrument to pull each specimen at a constant rate. That constant rate was programmed to correspond to 100% of the length of the floss per minute, as placed in the instrument after gripping. For example, if a specimen was 42 cm long, but after being placed into the instrument, the available length was 37 cm, the rate was programmed to 37 cm/min. This ensured that the elongation at break, which is calculated as a relative value versus the starting sample length, and its displacement at break, would always be accurate, irrespective of the variation in the original specimen length. The 34SC-2, aside from the force transducer, is also equipped with rotary encoders, so that it can track displacement with a resolution of 0.01 mm. Before mounting the utilized specimens on the tension testing system, a tempering step was carried out by removing them from refrigeration and allowing them to naturally reach room temperature over a period of 60 min.

Additionally, a cyclic load test was carried out by subjecting specimens of each of the four types studied by this work to variable forces, ranging from 50% to 90% of their maximum force at break, until the specimens finally broke, or until 100 cycles were reached. For this stage, a frequency of 0.2 Hz was selected, meaning that each sample would be loaded and relaxed once per five seconds. This was conducted to try and make an estimation of the fatigue effects on the flosses.

## 3. Results

Before constructing an experiment design, two limitations had to be taken into consideration. Firstly, the fact that every single floss user will use a different technique to floss, either due to different experience levels, different feelings of comfort, or owing to variable denture conditions in their oral cavity; therefore, even the same type of floss will generally experience different forces in both magnitude and orientation, variations in friction, and variations in bending. The second limitation is on the individual technique of the user. In general, for flosses in spools, a length of about 50 cm per use is suggested to be cut, with the majority of it being spooled around the fingers of the user; however, many users opt for longer or shorter lengths, as per their overall convenience.

### 3.1. Visual Observation of Untested Flosses

**Figure 3:**
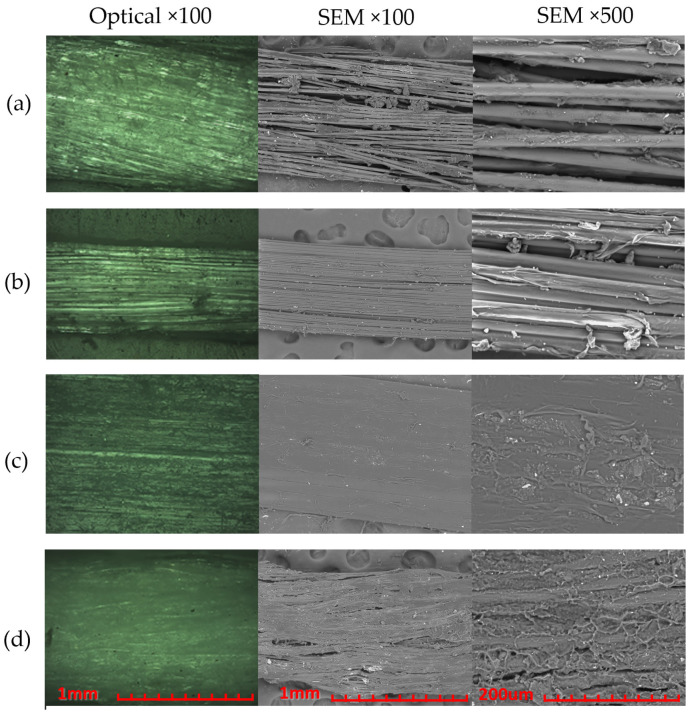
Optical and SEM images of untested flosses: (**a**) Lacalut; (**b**) Oral B Essential; (**c**) Oral B Satin; (**d**) Sensodyne.

Initially, a sample of each of the elected flosses was examined under SEM and optical microscopy, to determine a baseline of its structure, make, and potential defects. In Figure 3, optical microscopy and SEM images can be seen for an unused specimen of all flosses.

From the images at a magnification of ×100 it can be observed that (a) and (d) have a similar structure of long, twisted, cylindrical fibers of a comparable thickness. The ×500 magnification SEM images reveal that (d) is much more compacted than (a), potentially owing to the effects of waxing. Both (b) and (c), however, are shaped in a rectangular format, with more compacted fibers. This is most evident in (c) as its individual fibers have the smallest optical diameter among all flosses, and its cross-section is an elongated rectangle, even clearly visible to the naked eye.

### 3.2. Tension Testing of Flosses

**Figure 4:**
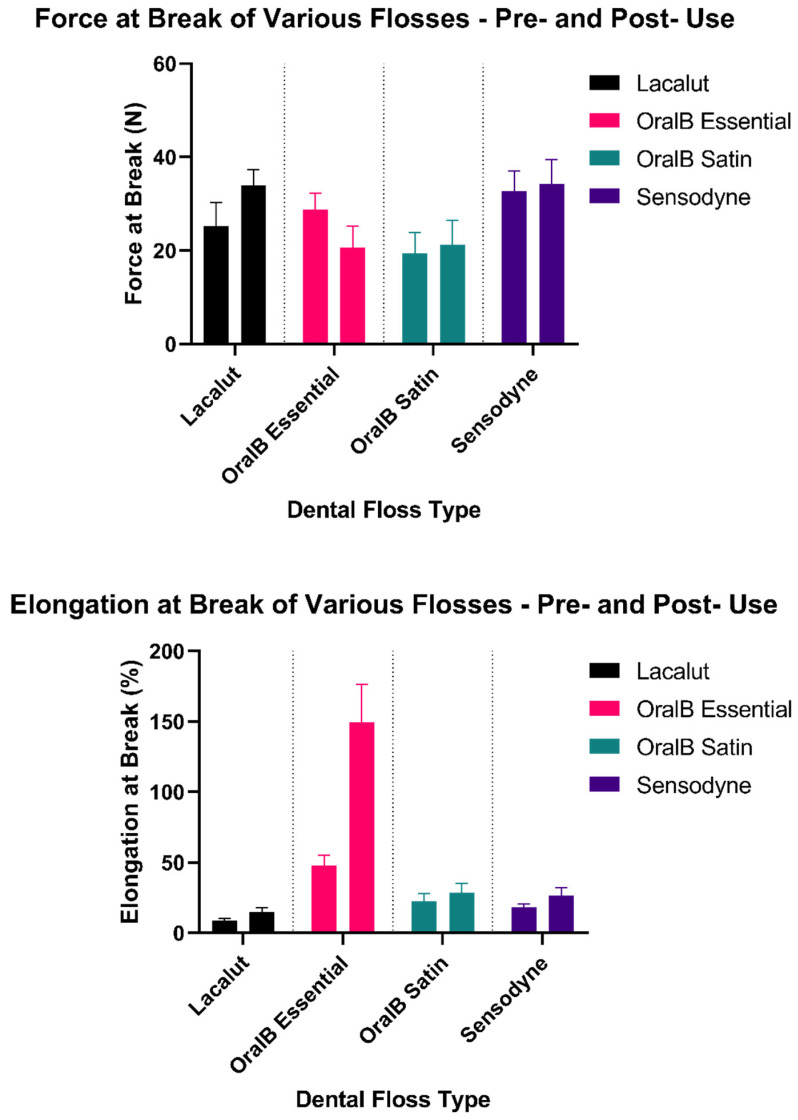
Force and elongation at break of dental flosses, mean values with standard deviation indicated. Left columns correspond to before and right columns to after usage.

For each of the four available flosses, ten volunteers were asked to use the product to clean their teeth and produce ten specimens. Each of these 400 specimens was subject to tension testing as per the method described above. Alongside these, 50 unused specimens of each of the flosses were also put to the test. Figure 4 presents the mean of the elongation at break and force at break along with the calculated standard deviation, for each of the flosses, both before (left columns) and after (right columns) utilization.

It is evident that in terms of maximum force at break, Oral B Satin and Sensodyne are the most stable flosses, failing at relatively comparable values, with almost the same standard deviations between the two cases. Lacalut fails at almost 10 N more pulling force post utilization, while Oral B essential has an almost reverse behavior, failing at about 10 N less. It is interesting to see that this opposing finding is also apparent in the standard deviation of the two, with the one of Lacalut slightly decreasing while the one of Oral B essential slightly increased.

However, the one notable result can be seen with the values for elongation at break. Immediately, a major instability can be seen in the case of Oral B Essential, which from an elongation of about 50% for non-used samples, skyrockets to an elongation of about 150% for used samples. This is a very important finding, as that is made possible only because the used floss, under the effect of the pulling force, starts to lose individual fibers as they start to snap. Even though that explains the seen increase in elongation, it is in principle an unfavorable property, as that means that slight imperfections in the user’s denture, technique, or handling while flossing, will cause the floss to partially break in the oral cavity. This can potentially lead not only to discomfort of the user, but also damages to the gums, or potential infection if fiber pieces become trapped below or around an implant or various dental restorations.

In general, before utilization, all mean values for forces at break are statistically significant between each other, ordered from highest to lowest as: Sensodyne > Oral B Essential > Lacalut > Oral B Satin. Significant statistical differences are present almost in every set; however, a notable difference is presented in the case of Oral B Essential, the elongation at break of which presents statistically significantly higher after use (*p* < 0.05, one-way ANOVA, Tukey post hoc test) while for all other flosses it presents statistically significantly lower.

**Figure 5:**
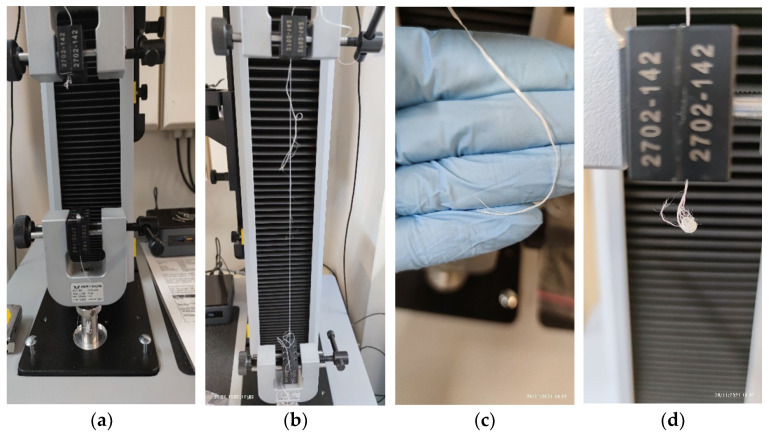
Breakage patterns of Lacalut (**a**), Oral B Essential (**b**), Oral B Satin (**c**), and Sensodyne (**d**).

Slight elongation increases can also be seen with Lacalut and Sensodyne, but Oral B Satin is found to be quite stable. Figure 5 demonstrates the breaking patterns of all four studied flosses.

In line with the statistical findings, (b) has a breaking behavior during which individual fibers start to fail until finally there is total separation. Oral B Satin (c) has a very clean cut at the breaking point, with only minor fiber separation, mostly owing to the very rapid tension release at break. Both Lacalut (a) and Sensodyne (d), also break relatively cleanly, with no individual fiber loss before total break; however, due to the very rapid tension release and their inertia, coupled with their wax coating, they form the pictured blobs on or close to the fixtures of the tension testing machine.

### 3.3. Fatigue Test of Flosses

**Figure 6:**
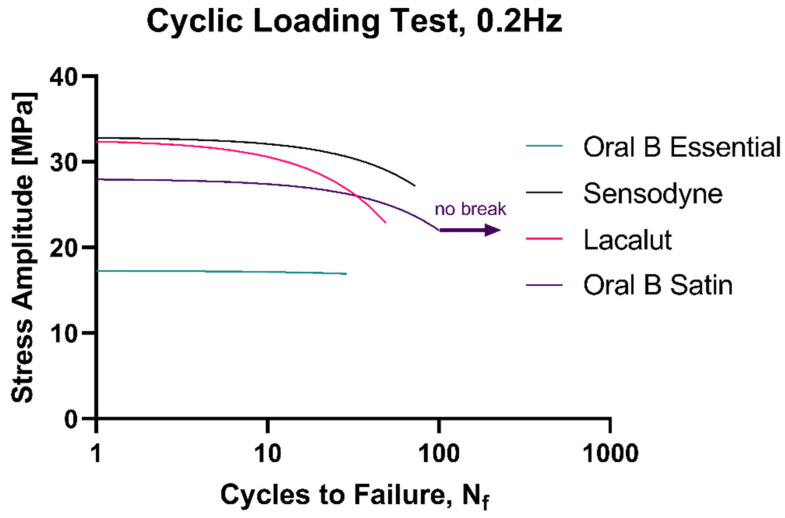
Cyclic loading response of the four studied flosses.

Healthy specimens of each of the flosses were subject to a cyclic loading test with a stop condition of either 100 cycles or the breaking point, if sooner. The test was carried out at a frequency of 0.2 Hz, so as to repeat one full load–unload cycle every 5 s. It can be observed that the Oral B Satin floss is the only one that withstood the full 100 cycles, probably owing to the fact that it is the only rectangular (tape) floss among the tested. Oral B Essential broke after the least cycles, but also had the smallest decrease in stress amplitude at break. Lacalut had a sharper profile of stress amplitude decrease versus Sensodyne, and it sustained 25 less cycles before failure (49 vs. 74) (Figure 6).

### 3.4. Visual Observation of Tested Flosses

**Figure 7:**
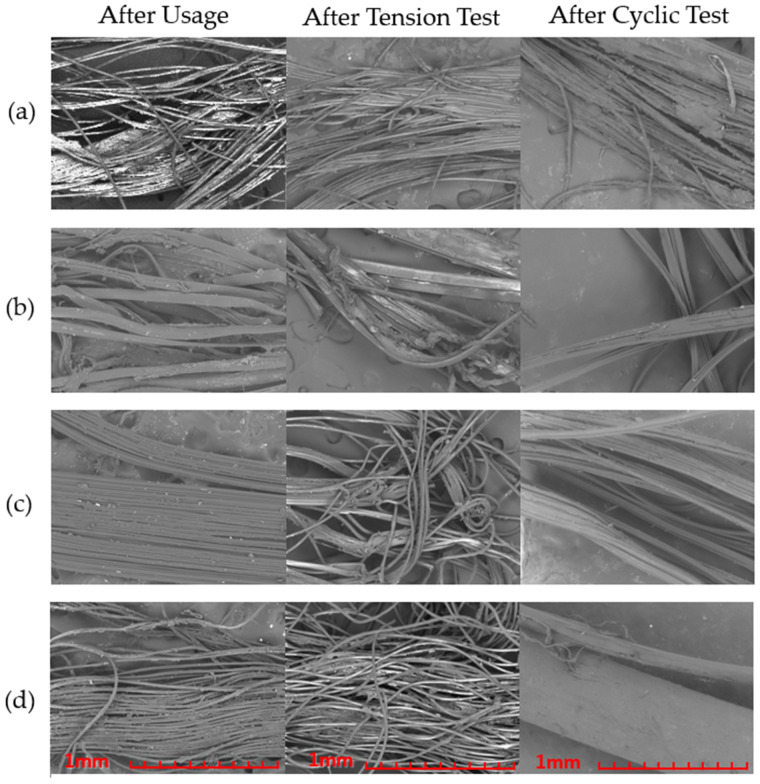
SEM images of floss defects of Lacalut (**a**), Oral B Essential (**b**), Oral B Satin (**c**), and Sensodyne (**d**) at an ×100 magnification.

After having performed the tension testing on all samples, scanning electron microscopy was repeated, this time to study the defects caused after breaking. In addition to that, utilized but non-tension-tested floss specimens were also examined to cross-reference which damage was caused by the force of breaking and which damage was caused under normal flossing (Figure 7).

Once again, a peculiar finding is about Oral B Essential (b), where even only after usage a lot of individual fibers can already be seen broken. This is of course only worsened after tension testing, with a very severe fiber entanglement and separation that bears no resemblance to the initial shape of the floss.

Oral B Satin can be seen transversely split after use, but it still retains its overall rectangular shape with some fibers twisted but almost none broken. After tension testing, it can also be seen that large portions have broken together, similar to Figure 5c.

Lastly, both Lacalut (a) and Sensodyne (d), have sustained minor visible structural damages, compared to their initial state in Figure 3a,d respectively, however, Sensodyne seems to have abolished most of its wax coating. After tension testing, they can both be seen partially tangled and their individual fibers more curled than before, but that wavy pattern can partially be attributed to the ripple effect at the point of break, where the tensioned fibers were all acting similar to dampers leading to their rapid untwisting and moving towards their anchor points at the same time, which translated to this plastic deformation.

With respect to the cyclic testing, it can be seen that Sensodyne demonstrates the most different behavior versus the tensile test, retaining most of its original shape. Oral B Satin, as expected, is quite similar to the after-use case, as it did not break, while the other two flosses have damage between the after usage and after-tension test levels.

## 4. Discussion

**Figure 8:**
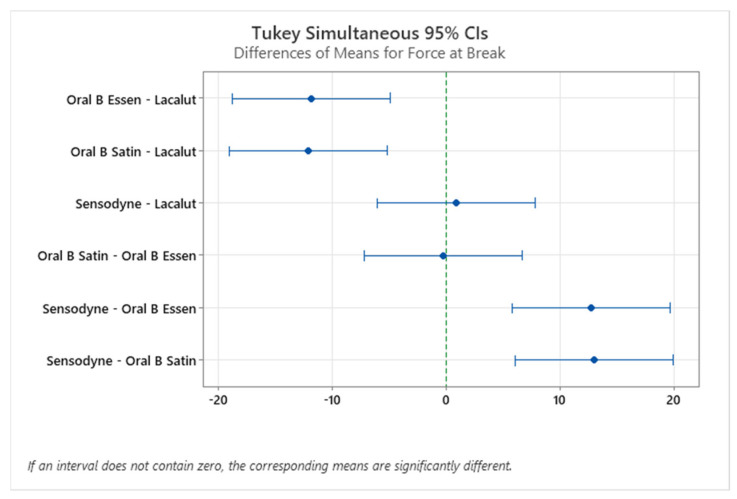
Differences of means for force at break, Tukey HSD test.

**Figure 9:**
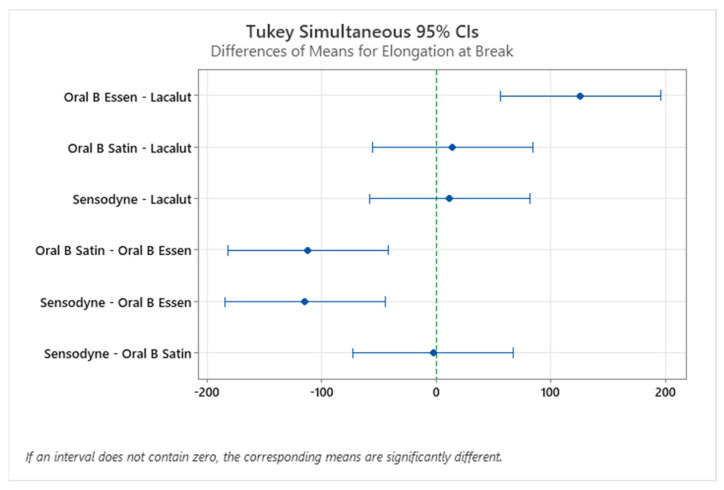
Differences of means for elongation at break, Tukey HSD test.

An interesting point in the study would be a more in-depth statistical analysis of the experimental values. Figure 8 and Figure 9 show the Tukey HSD (honestly significant difference) test interval plot for differences of means for the force at break and elongation at break, respectively. It can be seen that only the difference between the means of Sensodyne and Lacalut and Oral B Satin and Oral B Essential are not statistically significant in the case of force at break, while in the case of elongation at the break, the non-statistically significant mean differences can be found between Oral B Satin–Lacalut, Sensodyne–Lacalut, and Sensodyne–Oral B Satin.

**Figure 10:**
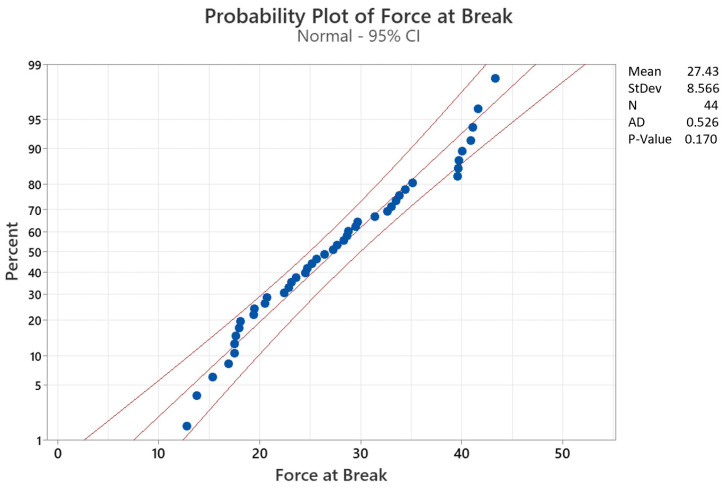
Normal distribution probability plot for force at break.

**Figure 11:**
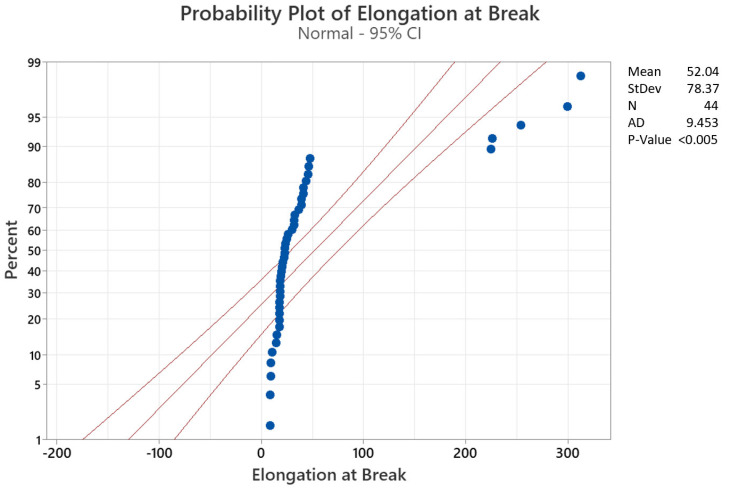
Normal distribution probability plot for elongation at break.

In Figure 10 and Figure 11, the probability plots for the two parameters are displayed. It can be easily observed that while the force at break follows quite closely a normal distribution, that is not the case with the elongation at break, where five outliers can clearly be seen. Even though it is an expected result, due to several occurrences of partial breaks of floss specimens among all volunteers and/or unused specimens, the peculiarity is that each of the five outliers corresponds to the Oral B Essential floss of five distinct volunteers. This result, however, also clearly demonstrates that the overall degradation of the floss is quite unpredictable, and the need for machine learning models is crucial in order to create adequate predictions for personalized suggestions of flosses based on the individual needs and conditions of each subject.

## 5. Conclusions

The scope of this work has been to study any performance alterations of commercially available dental flosses after they have been used. Due to the steady growth in the use of flosses, and the introduction of new materials and construction methods, a rise in the number of reported occurrences of discomfort from users has been recorded. This prevents flosses from becoming a universal dental cleaning tool, and mandates clinicians to further study the material properties of individual flosses to provide better suggestions.

This study has showcased significant performance degradation of dental flosses after they have been used. This was realized in vitro, after ten volunteers used ten specimens each of the four studied flosses to floss normally. Most notably, the mean elongation at break of the Oral B Essential floss varied from 50% to 150% after use, which coupled with visual evidence of partial breakage potentially calls for specific utilization instructions to be given to patients on a case-by-case basis by their dental practitioner. Oral B Satin overall was a much more stable candidate, while Lacalut also experienced a rise in sustainable force at break and elongation, again indicative of partial breaks.

Therefore, it is of crucial importance to perform such experimentation in a larger scale, not only to study the effects of complex clinical cases, (such as prosthetics, implants, and orthodontic abnormalities on flosses, but also to potentially, with the aid of machine learning models and artificial intelligence to study and construct materials for flosses, which in the near future may form tailored suggestions and be personalized to the needs of every individual.

## Data Availability

Study data are available from the authors upon request, however no identifying information on the study participants will be made available, in line with the applicable data protection regulation laws and ethics guidelines.
